# Molecular point-of-care: technological evolution, clinical applications, and future perspectives

**DOI:** 10.3389/fbioe.2026.1908277

**Published:** 2026-09-25

**Authors:** Danfeng Zhang, Xin Cui, Ruiling Zhang, Wenlong Cai, Li Wang, Jichun Yang

**Affiliations:** 1 School of Biomedical and Chemical Engineering, Liaoning Institute of Science Technology, Benxi, China; 2 Department of Chemistry, College of Sciences, Northeastern University, Shenyang, China; 3 School of Medicine, Chongqing University, Chongqing, China; 4 Department of Clinical Laboratory, Henan Provinve Engineering Technology Research Center of Rapid-Accuracy Medical Diagnostics, The First Affiliated Hospital of Henan University, Henan University, Kaifeng, China

**Keywords:** clinical diagnostics, microfluidic chips, molecular techniques, point-of-care testing, precise healthcare

## Abstract

The landscape of clinical diagnostics is undergoing a fundamental shift from centralized laboratories toward decentralized point-of-care testing (POCT) models. Conventional molecular diagnostics often suffer from prolonged turnaround times, high operational costs, and a strict reliance on sophisticated infrastructure. To overcome these limitations, modern POCT platforms have deeply integrated advanced molecular techniques. These include diverse isothermal nucleic acid amplification methods (such as LAMP, RPA, and RCA) and highly specific CRISPR/Cas recognition systems. Furthermore, coupling these biochemical reactions with microfluidic chips and smartphone-based digital platforms has enabled fully automated, “sample-in, answer-out” diagnostic capabilities. This review systematically summarizes the technological evolution of these molecular POCT platforms. It highlights their transformative clinical applications across diverse fields, including infectious disease containment, early oncology screening, cardiovascular emergency triage, and metabolic disease management. Despite remarkable progress, several critical bottlenecks still prevent the widespread clinical translation of molecular POCT. These challenges include cross-interference in multiplexed detection, enzyme stability outside strict cold chains, high costs of core components, and the lack of tailored regulatory frameworks. Addressing these barriers via synthetic biology, robust system integration, and artificial intelligence is critical to advancing the core vision of delivering rapid, accessible, and precise healthcare solutions in resource-limited settings.

## 1 Introduction

The landscape of clinical diagnostics is experiencing a notable paradigm shift, transitioning from centralized, infrastructure-heavy laboratories toward decentralized, patient-centric models (Vashist et al., 2015). Traditional molecular diagnostics, although highly accurate, are often limited by prolonged turnaround times (TAT), reliance on complex thermal cycling equipment, and substantial operational costs (Adamson et al., 2020). Molecular POCT has emerged as a crucial component of this transition. By facilitating rapid sample processing, localized nucleic acid amplification, and timely signal readout, POCT supports more immediate clinical decision-making across primary healthcare settings, outpatient clinics, and resource-limited regions (Collinson, 2020; Khodakov et al., 2021).

Recent global health challenges, such as the COVID-19 and mpox outbreaks, have highlighted the utility of POCT for rapid pathogen identification, which is essential for interrupting transmission chains (Vashist, 2020). Beyond infectious diseases, the application of POCT is increasingly expanding into areas like early cancer screening and chronic disease management (Lakhera et al., 2025; Zhao L. et al., 2023). To address these broadening clinical needs and the public shift toward proactive health management, molecular POCT technologies have evolved significantly (Li et al., 2026). Innovations in isothermal amplification technologies (IATs), including LAMP and RPA, have largely mitigated the thermal cycling requirements of conventional PCR (Li et al., 2025; Zu et al., 2025). Additionally, the integration of CRISPR/Cas systems has improved target specificity, often enabling single-nucleotide resolution. Concurrently, advancements in microfluidics and smartphone-based readout platforms have contributed to improved automation, higher throughput, and enhanced data integration in these assays (Li et al., 2024; Miron et al., 2024).

In light of these advancements, this review provides a systematic overview of the current technological trajectories in modern molecular POCT. Figure 1 provides a schematic overview of the central contents of molecular POCT covered in this review. We evaluate the underlying principles of these platforms, with a specific focus on the synergistic integration of various nucleic acid amplification methods and microfluidic systems. Furthermore, we summarize their emerging applications across diverse clinical disciplines, including infectious diseases, oncology, and cardiovascular care. Finally, this review discusses current technological and translational challenges while outlining potential future development trends. Through this analysis, we aim to offer a balanced perspective that can inform ongoing scientific research, technological refinement, and clinical implementation in this rapidly evolving field.

**FIGURE 1:**
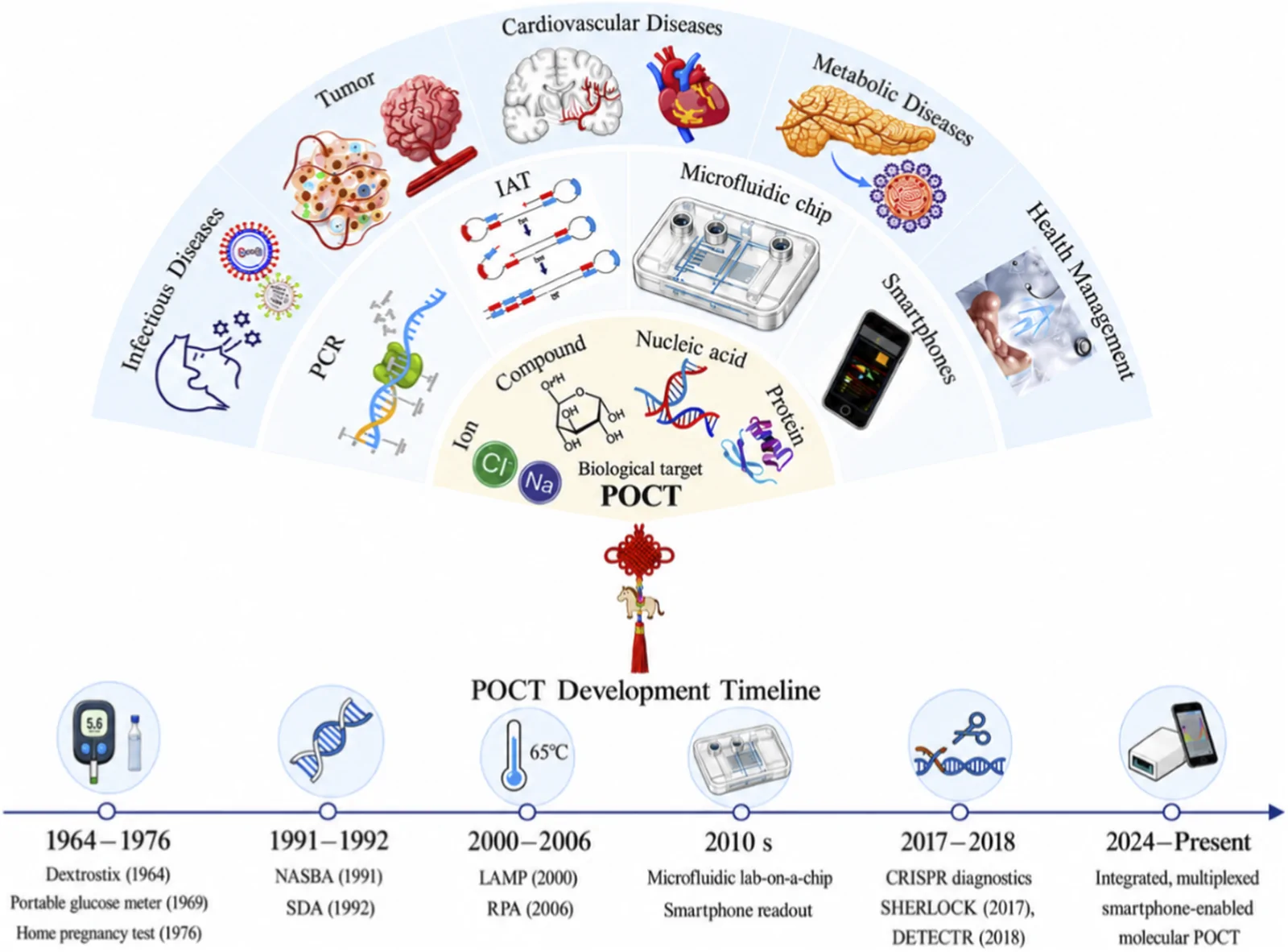
Main technologies, application fields and development timeline of molecular POCT.

## 2 Molecular diagnostic techniques: the analytical engine of POCT

Molecular diagnostic techniques provide a foundational framework by targeting key biomarkers, primarily nucleic acids (DNA, RNA) and specific proteins. Through core mechanisms such as molecular recognition and signal amplification, these platforms facilitate the qualitative and quantitative analysis of pathogenic microorganisms and genetic mutations. Notably, while traditional PCR-based strategies remain central to this field, the technological portfolio has increasingly broadened to incorporate a variety of isothermal and signal amplification methodologies (Yin et al., 2026).

Concurrently, POCT serves a vital role in near-patient clinical care and public health responses, owing to its capacity for on-site sampling and rapid result delivery. However, conventional POCT platforms frequently encounter challenges in maintaining high analytical specificity within complex sample matrices or achieving multiplexed detection. To address these limitations, the integration of advanced molecular diagnostics offers promising solutions, providing enhanced sensitivity, specificity, and adaptability for platform integration.

The development of molecular POCT has unfolded across three distinct generations over the past 3 decades. The first generation (1990 s–2000 s) featured miniaturized PCR systems and early isothermal technologies (NASBA, SDA) that first reduced dependence on large thermal cyclers (Zhao et al., 2015). The second generation (2010 s) witnessed the mainstream adoption of LAMP and RPA, alongside the breakthrough application of CRISPR/Cas systems for diagnostics, which dramatically improved specificity without increasing instrument complexity. The third generation (2020 s - present) is defined by fully integrated sample-to-answer microfluidic systems, smartphone-enabled readouts, and broad multiplexing capability, accelerated exponentially by the COVID-19 pandemic. This trajectory reflects a consistent push toward simpler operation, higher analytical performance, and equitable global access.

### 2.1 PCR: the gold standard for high-sensitivity amplification

POCT facilitates rapid decentralized clinical decision-making. However, conventional immunoassay-based POCT often faces inherent limitations, such as restricted sensitivity, elevated false-negative rates for low-abundance targets, and susceptibility to non-specific false positives, particularly in resource-limited settings (Ismadi et al., 2024). PCR remains a gold standard for nucleic acid detection, offering high sensitivity, often approaching the single-copy level, and robust specificity through sequence-specific amplification (Saiki et al., 1988). Recent technological advances have largely mitigated its traditional drawbacks, such as bulky instrumentation and prolonged turnaround times, thereby facilitating its integration into POCT platforms (Jebbink et al., 2003; Zhang S. et al., 2026).

For instance, Lu et al. integrated plasmonic thermal cycling with visual detection on a single PCR test strip (Figure 2a), achieving a limit of detection (LOD) of one copy/µL for the influenza A virus within 15 min (Lu et al., 2025). This approach helps balance operational convenience with diagnostic reliability for low-abundance targets. Furthermore, the PCR-based design reduces the risk of false positives resulting from homologous pathogen cross-reactivity, a common limitation in non-amplification POCT methods. Additionally, a digital acoustofluidic rapid PCR system developed by Huang et al. employs acoustic technology to enhance amplification efficiency (Figure 2b) (Huang et al., 2025). While maintaining high sensitivity, this system shortens the total assay time to 57 min. It enables the direct analysis of unprocessed whole blood with an LOD of 0.5 ng/mL, substantially reducing the need for complex sample pre-treatment.

**FIGURE 2:**
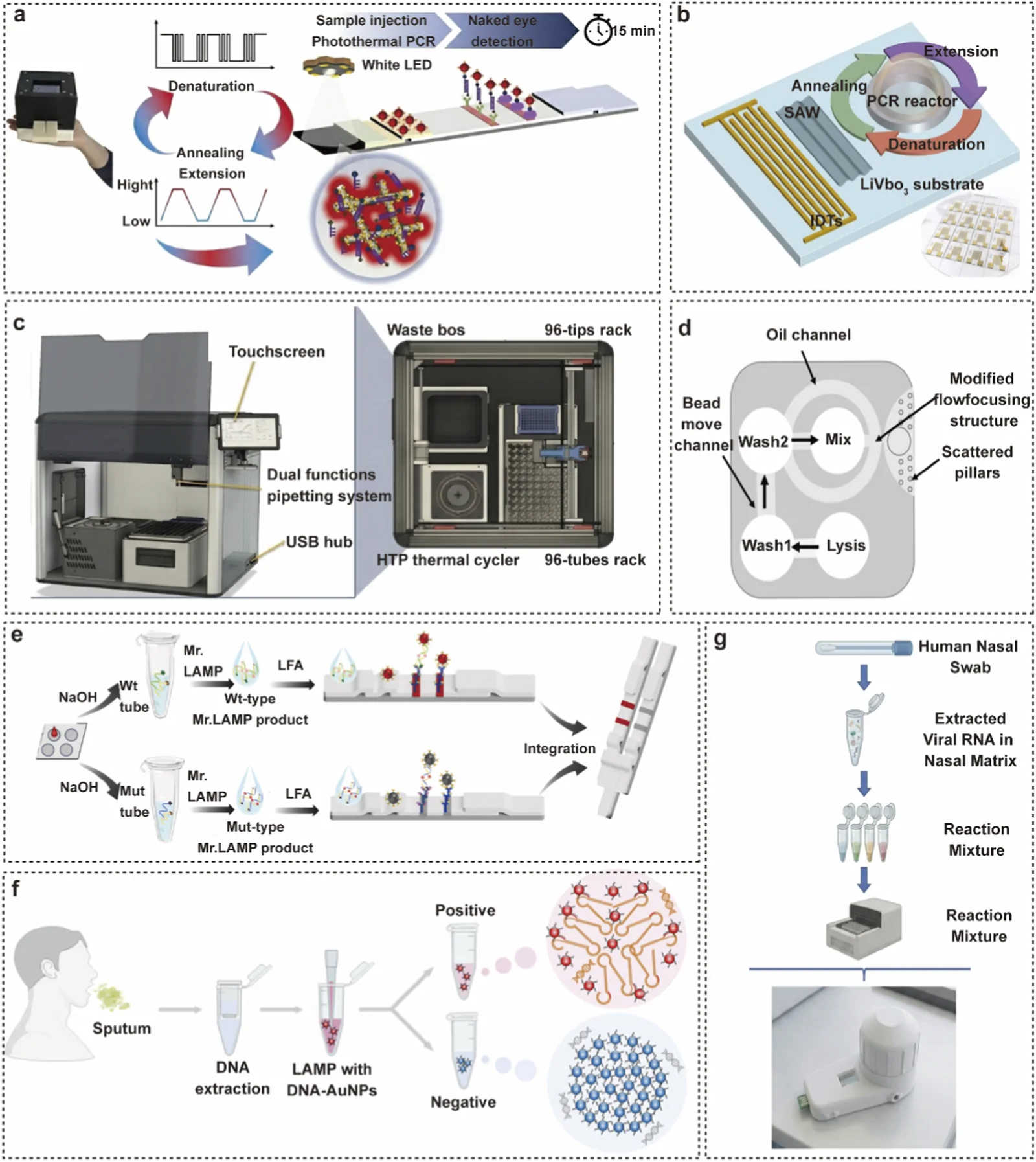
**(a)** Schematic of volumetric heating utilizing broadband-absorbing plasmonic fabric for a PCR test strip (Lu et al., 2025). **(b)** A digital acoustofluidic on-chip PCR system (Huang et al., 2025). **(c)** The high-throughput molecular diagnostic platform (Phan et al., 2026). **(d)** Detailed view of the PDMS (polydimethylsiloxane) microstructures (Ma et al., 2023). **(e)** Lateral flow assay readout of the Mr-LAMP amplification products (Wang S. et al., 2026). **(f)** Schematic illustration of an *in vitro* diagnostic platform for tuberculosis based on a naked-eye LAMP assay for *M. tuberculosis* (Zhang X. et al., 2024). **(g)** The multiplexed point-of-care nucleic acid diagnostic platform (Jiang et al., 2024).

Clinical diagnostic scenarios frequently necessitate the simultaneous detection of multiple pathogens that present with similar symptoms, thereby requiring robust multiplexing capabilities. Conventional multiplex POCT methods often encounter primer cross-interference and compromised sensitivity. To address these issues, Phan et al. developed a switching-PCR integrated centrifugal microfluidic system (Figure 2c), which enables the fully automated detection of 36 target genes across 6 parallel samples in a single run (Phan et al., 2026).

Furthermore, the precise quantification of pathogen load is valuable for disease staging and therapeutic monitoring. Digital PCR (dPCR) (Vogelstein and Kinzler, 1999) facilitates absolute quantification with improved tolerance to inhibitors, circumventing the need for standard calibration curves (Figure 2d) (Ma et al., 2023). For example, Ma et al. developed an integrated droplet digital PCR (ddPCR) microfluidic chip that provides battery-powered, palm-sized absolute quantification with an LOD of 10 copies/µL (Figure 2e) (Wang S. et al., 2026). Despite these advancements, PCR-based POCT continues to face certain challenges, including the cost of disposable consumables and the limited room-temperature stability of reagents. Consequently, future research is likely to focus on the development of lyophilized reagents and further system miniaturization.

### 2.2 Isothermal amplification technologies (IATs): eliminating thermal cycling

Unlike conventional PCR, which relies on multi-temperature cycling, IATs represent a category of nucleic acid amplification methods that eliminate stepwise temperature regulation. By operating at a single constant temperature, IATs enable efficient target amplification through specialized enzyme systems (Pyrc et al., 2014; Jaroenram et al., 2020). By circumventing the thermal cycling required by conventional PCR, IATs address the infrastructural limitation of complex thermal control, reducing equipment barriers and operational complexity for decentralized testing. While PCR exhibits absolute quantification and single-copy detection sensitivity suitable for high-precision clinical laboratories, IATs offer advantages in energy efficiency and device miniaturization. Furthermore, when IATs are coupled with CRISPR-based diagnostics and integrated POCT microfluidic systems, they form an analytical framework that balances analytical sensitivity with on-site deployability. However, a persistent challenge across most IATs remains their susceptibility to non-specific amplification, which necessitates careful optimization in multiplexed scenarios. Clinically, IAT-driven POCT is primarily suited for resource-limited settings, rapid triage in outpatient clinics, and immediate public health responses where rapid, infrastructure-independent screening is prioritized. In general, IATs shift molecular diagnostics toward field-deployable formats by trading the precision of thermal cycling for operational simplicity and speed.

#### 2.2.1 LAMP: rapid and robust signal enrichment

Loop-mediated isothermal amplification (LAMP) enables highly efficient nucleic acid amplification under constant temperature conditions, typically ranging between 60 °C and 65 °C. This technique achieves an exponential 10^9^-to 10^10^-fold amplification within a rapid timeframe of 15–30 min (Notomi et al., 2000). Moreover, LAMP demonstrates a robust tolerance to biological matrix inhibitors. Leveraging this favorable property, Zhang et al. developed an extraction-free, visual LAMP platform for the direct detection of *Mycobacterium tuberculosis* in unprocessed sputum samples (Figure 2f) (Zhang X. et al., 2024). By circumventing conventional centrifugation and nucleic acid purification steps, this system completes the assay within 50 min with a limit of detection (LOD) of 10 CFU/mL. Further, a multiplex reverse transcription-LAMP (RT-LAMP) paper-based platform was established by Yager’s group for the parallel detection of multiple respiratory RNA viruses to address the clinical requirements of syndromic diagnostics (Figure 2g) (Jiang et al., 2024). Compared to traditional PCR, LAMP accelerates diagnostic workflows and exhibits resilience against crude sample inhibitors, addressing the limitation of tedious pre-analytical purifications. However, its broader clinical deployment is hindered by unresolved challenges, such as the necessity for 4–6 primers per target makes high-order multiplexing complex, and open-tube formats pose a risk of aerosol contamination. Additionally, unlike CRISPR-integrated systems, standalone LAMP has difficulty achieving single-nucleotide resolution. LAMP is generally applied in single-target or low-plex rapid screening scenarios of primary-level pathogen detection and food safety control, where speed and simplicity are prioritized (Kasputis et al., 2024). Ultimately, LAMP functions as a robust amplification tool for crude matrices, though its multiplexing limitations restrict its utility in complex panel diagnostics.

#### 2.2.2 RPA: near-physiological temperature operations

Recombinase polymerase amplification (RPA) operates through the coordinated action of recombinases, single-stranded DNA-binding proteins (SSBs), and polymerases, facilitating rapid nucleic acid amplification at mild constant temperatures, typically between 37 °C and 42 °C (Piepenburg et al., 2006). By operating near physiological temperatures, RPA addresses the limitation of high-temperature heating required by LAMP and PCR, offering the advantage of utilizing passive heat sources (e.g., chemical warmers). Compared to LAMP, RPA generally requires less complex primer designs and lower incubation temperatures, which further minimizes equipment dependency. However, conventional RPA often encounters challenges such as non - specific amplification and limited multiplexing capabilities within a single homogeneous reaction. To address these bottlenecks, recent research has focused on integrating RPA with closed-system designs, alternative heat sources, and spatial compartmentalization strategies. For instance, a closed-system mpox virus diagnostic kit was engineered to mitigate the pervasive risk of aerosol contamination, (Figure 3a) (Yu J. et al., 2025), and Li et al. developed a self-contained microfluidic system to further reduce the infrastructure reliance for SARS-CoV-2 detection by integrating reverse transcription-RPA (RT-RPA), CRISPR-Cas12a, and lateral flow (Figure 3b) (Li Z. et al., 2022). Moreover, the characteristic of rapid amplification at room temperature of RPA provides convenient conditions for the combination with other technologies, which can efficiently resolve the inherent multiplexing limitations of RPA. For the simultaneous detection of multiple targets, a MiCaR platform was introduced by coupling microfluidic spatial coding with multiplex RPA and CRISPR - Cas12a cleavage, this platform achieves the simultaneous detection of up to 30 nucleic acid targets using a single universal fluorescent probe, thereby significantly expanding the diagnostic utility of RPA (Figure 3c) (Xu Z. et al., 2022). Despite these developments, the cost of multi-enzyme reagents and the risk of contamination upon lid removal remain unresolved challenges. Clinically, RPA-especially when coupled with CRISPR sensors to ensure specificity, is suited for emerging infectious disease surveillance and remote POCT in resource-limited environments. In conclusion, RPA offers high field-adaptability among current IATs due to its minimal thermal requirements, functioning well when integrated with CRISPR or microfluidics to overcome its inherent specificity limitations.

**FIGURE 3:**
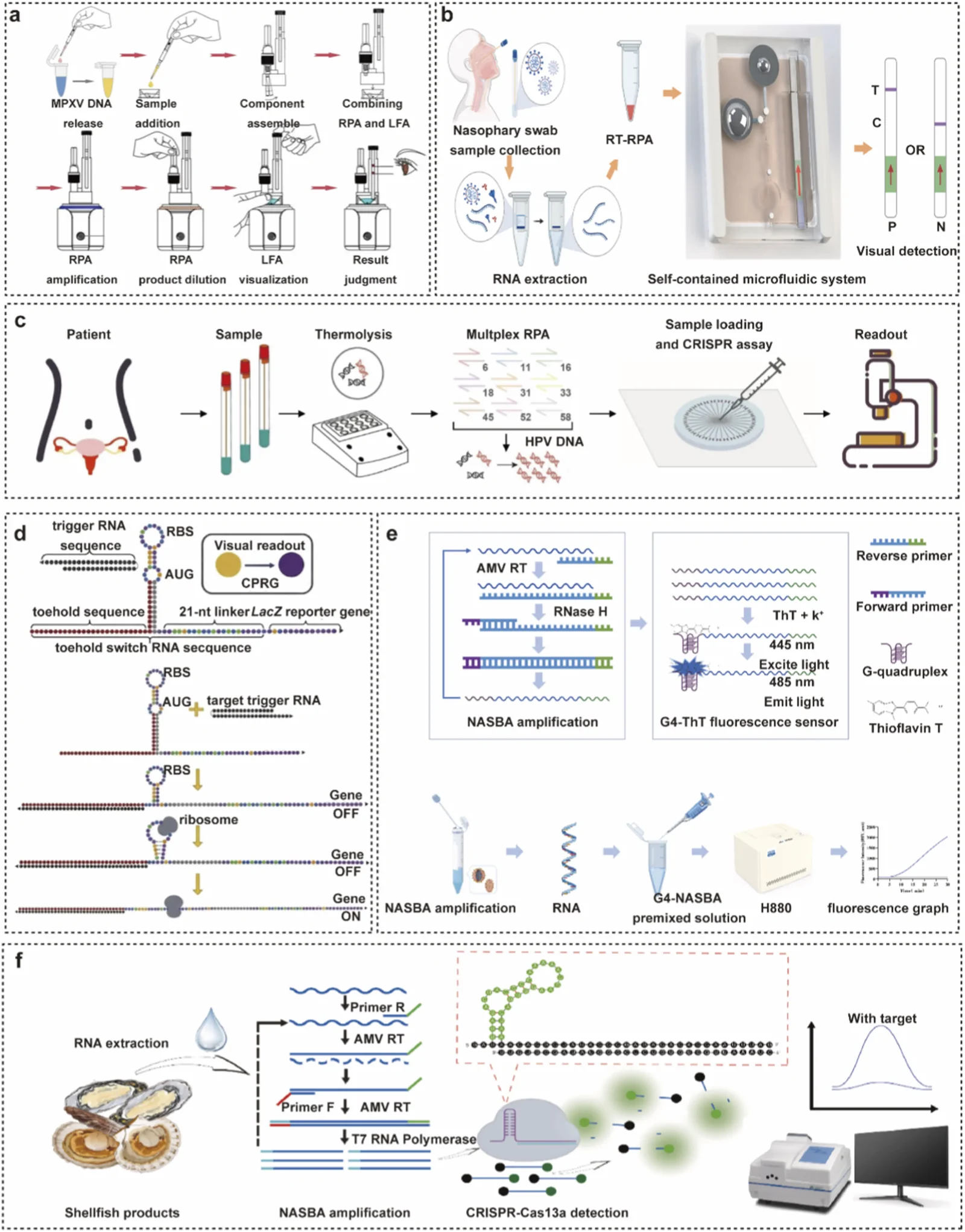
**(a)** Schematic illustration of the LFA-RPA POCT kit workflow for mpox virus detection (Yu J. et al., 2025). **(b)** Experimental workflow for SARS-CoV-2 detection with the self-contained microfluidic system (Li Z. et al., 2022). **(c)** The MiCaR platform integrated with the microfluidic device of CRISPR-Cas12a and multiplex RPA (Xu Z. et al., 2022). **(d)** The design and regulatory mechanism of a colorimetric toehold switch sensor (Cao et al., 2021). **(e)** Schematic workflow of the G4-modified nucleic acid sequence-based amplification assay (Luo et al., 2026). **(f)** Working principle of the NASBA/Cas13a-mediated detection system (Mao et al., 2024).

#### 2.2.3 NASBA: direct targeting of RNA pathogens

Nucleic Acid Sequence-Based Amplification (NASBA) is an isothermal amplification technique tailored specifically for RNA targets. Operating at a mild, constant temperature of approximately 41 °C, it relies on the coordinated action of avian myeloblastosis virus (AMV) reverse transcriptase, ribonuclease H (RNase H), and T7 RNA polymerase (Compton, 1991). NASBA circumvents the need for a separate reverse transcription phase, addressing the multi-step complexities inherent in standard RT-PCR. This characteristic for continuous RNA-to-RNA amplification provides a streamlined workflow that is advantageous for RNA viral detection. To facilitate point-of-care applications, an instrument-free, paper-based NASBA platform was constructed by Wang’s team for the visual detection of respiratory syncytial virus (RSV) (Figure 3d) (Cao et al., 2021), and Luo et al. reported an ultra-fast G4-NASBA system coupled with a portable fluorescence sensor for influenza A virus detection, which also significantly enhanced the sensitivity of the detection via NASBA method (Figure 3e) (Luo et al., 2026). Through the optimization of enzymatic reaction kinetics, this system achieves specific amplification within 15 min. Additionally, to overcome complex matrix interference, Kim et al. combined NASBA with Cas13a cleavage for the detection of human norovirus in shellfish samples (Figure 3f) (Mao et al., 2024). Nevertheless, the broader clinical utility of NASBA is constrained by an unresolved issue, which is that its three-enzyme system requires control over pH and ionic concentrations, making it sensitive to matrix inhibitors. Therefore, NASBA is less adaptable to crude, unprocessed specimens compared to RT-LAMP or RT-RPA. Clinically, it remains a specialized tool applicable in targeted RNA detection applications, such as viral load monitoring and gene expression analysis, provided that adequate sample preparation is implemented. Although NASBA provides a direct route for RNA pathogen amplification, its environmental sensitivity necessitates integration with upstream purification to realize its diagnostic potential.

#### 2.2.4 RCA: exceptional signal-to-noise ratios

Rolling circle amplification (RCA) employs circular single-stranded DNA as a template for continuous replication. Driven by high-fidelity, strand-displacing DNA polymerases, this reaction proceeds under mild isothermal conditions (typically 30 °C–37 °C), continuously yielding long single-stranded DNA products with tandem repeat sequences (Fire and Xu, 1995). By generating repetitive amplicons from a single recognition event, RCA addresses the limitation of high background interference observed in other isothermal techniques. Its primary advantage over PCR and LAMP is its favorable signal-to-noise ratio, conferring signal amplification without thermal denaturation. To leverage these capabilities in clinical diagnostics, various integrated POCT platforms have been explored (Choi et al., 2022). Caneira et al. designed a microfluidic padlock probe (PLP)-RCA assay that incorporates solid-phase capture and on-chip fluorescence detection. The implement of RCA on a microfluidic chip ensured both the sensitivity and specificity of the pathogen detection at the same time. (Figure 4a) (Caneira et al., 2025). However The combination of multiple modules in a POCT system also complicated the process of condition optimization and detection. On this basis, Jain et al. integrated PLP-RCA with nuclease protection and lateral flow readouts to create the PLAN-LFA system for rapid and simple genetic testing, which further streamline the operational workflow (Figure 4b) (Jain et al., 2021). Expanding the diagnostic utility of RCA beyond nucleic acid targets, Chen et al. established an RCA-assisted microfluidic immunosensing platform tailored for bedside allergy diagnostics (Figure 4c) (Chen et al., 2024). By coupling this system with smartphone-based imaging, it enables the multiplexed profiling of allergen-specific IgE directly in unprocessed whole blood, highlighting the broad adaptability of RCA in decentralized clinical settings. Nevertheless, unresolved challenges, such as prolonged reaction durations (often 60–120 min) and the dependency on circular probe designs, prevent its widespread use as a standalone rapid POCT tool. Therefore, in clinical scenarios, RCA is predominantly applied as a downstream signal amplification module for low - abundance biomarkers, SNP analysis, and protein quantification. To summarize, RCA is a valuable asset for specificity signal enhancement, though its slow kinetics dictate its role as a supportive amplification layer rather than a frontline rapid diagnostic method.

**FIGURE 4:**
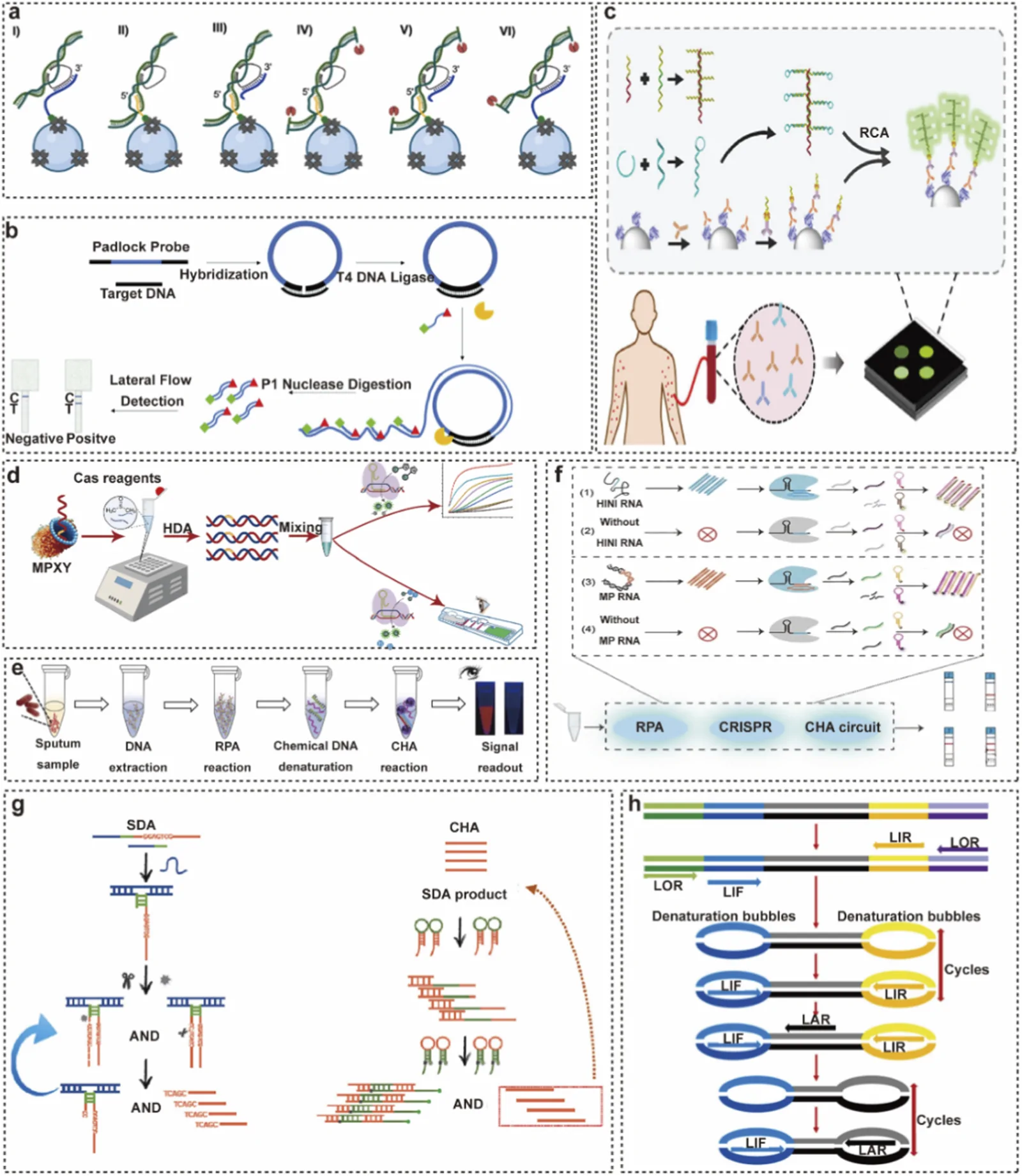
**(a)** Schematic workflow of PLP-RCA process (Caneira et al., 2025). **(b)** Schematic representation of padlock RCA and the nuclease-assisted PLAN-LFA (Jain et al., 2021). **(c)** Multiplex analysis strategy on the mcMAD platform for sIgE detection (Chen et al., 2024). **(d)** Schematic of a DMSO-enhanced, one-pot HDA-CRISPR/Cas12a biosensor. **(e)** The RPA-CHA-assisted dual signal amplification strategy for the visual detection of tuberculosis (Hu et al., 2024). **(f)** The LFA platform utilizing a triple cascade amplification system for M. pneumoniae (MP) DNA and H1N1 RNA detection (Wei et al., 2025). **(g)** Schematic illustration and working principle of the TWJ-mediated SDA-cascade-CHA system for tumor-associated miRNAs detection (Yao et al., 2025). **(h)** Working principle of the LDIA method (Gou et al., 2023).

#### 2.2.5 HDA: mimicking *in vivo* DNA replication

Helicase-dependent amplification (HDA) is an isothermal amplification technique that mimics the natural mechanism of *in vivo* DNA replication. Operating at temperatures between 37 °C and 65 °C, this biomimetic approach relies on the coordinated action of a DNA helicase, single-stranded DNA-binding proteins (SSBs), and a strand-displacing DNA polymerase (Vincent et al., 2004). HDA resolves the complex primer design barriers inherent in LAMP, offering the advantage of operating with a standard pair of linear primers, similar to traditional PCR. To harness this operational simplicity for POCT, A DMSO-enhanced HDA coupled with a CRISPR/Cas12a lateral flow biosensor was constructed by Yu et al. for the rapid detection of the mpox virus (Figure 4d) (Yu L. et al., 2025). Despite its conceptual simplicity, the broader commercialization of HDA remains constrained by unresolved challenges, chiefly the limited thermostability of wild-type helicases, sequence-dependent efficiency variations, and core enzyme production costs. Consequently, HDA currently occupies a niche position and is clinically suited for low-abundance clinical screening and specific integrated pathogen POCT platforms, where primer simplicity outweighs reagent expenses (Liao et al., 2026). In brief, while HDA mimics natural replication to simplify assay design, protein engineering breakthroughs are required to improve enzyme stability and reduce costs before it can compete with more established IATs.

#### 2.2.6 CHA: enzyme-free exponential signal amplification

Catalytic hairpin assembly (CHA) is an enzyme-free isothermal signal amplification strategy typically initiated by a specific target nucleic acid sequence. Rather than relying on polymerases, the target catalyzes a thermodynamically driven, cyclic self-assembly cascade among metastable hairpin DNA probes (Yin et al., 2008). By operating without enzymes, CHA solves the limitation of cold-chain dependency associated with PCR and other protein-based IATs, offering the advantage of room-temperature storage. To leverage these advantages in clinical diagnostics, researchers have increasingly integrated CHA with upstream pre-amplification techniques. For instance, Hu et al. developed an RPA-CHA cascade platform for *Mycobacterium tuberculosis* detection (Figure 4e) (Hu et al., 2024). By utilizing CHA as a downstream amplifier, this approach improves diagnostic sensitivity for low-abundance pathogens while reducing the cold-chain dependence associated with complex multi-enzyme systems. Furthermore, Wei et al. reported an RPA-CRISPR/Cas12a-CHA triple-cascade lateral flow platform for the simultaneous detection of *Mycoplasma pneumoniae* and the H1N1 influenza virus directly in unprocessed saliva (Figure 4f) (Wei et al., 2025). Even so, unresolved challenges impede its translation into clinical practice, among which are relatively slow reaction kinetics and background-noise generation caused by spontaneous, target-independent dissociation of hairpin probes. Due to these concentration-dependent constraints, CHA is clinically most applicable not as a standalone test, but as a secondary signal booster for infectious diseases and tumor markers when cascaded with upstream enzymatic methods (Liu et al., 2025a). In essence, CHA provides a stable, enzyme-free amplification alternative, serving as a synergistic signal enhancer within multi-stage diagnostic cascades.

#### 2.2.7 SDA: a dual recognition mechanism

Strand displacement amplification (SDA) relies on the coordinated action of a restriction endonuclease, typically functioning as a nicking enzyme, and a strand-displacing DNA polymerase. Operating under mild isothermal conditions, usually between 37 °C and 41 °C, this technique achieves efficient nucleic acid amplification through continuous cycles of single-strand nicking and subsequent polymerase-driven strand displacement (Walker et al., 1992). SDA addresses the challenge of non-specific cross-reactivity seen in single-recognition assays. Its inherent dual-recognition mechanism, which mandates both specific target hybridization and precise restriction site recognition, confers a target discrimination capability that is generally more robust than less constrained isothermal methodologies. For example, an SDA-CHA cascade strategy was designed to evaluate microRNA (miRNA) expression levels in tumor cells (Figure 4g) (Yao et al., 2025). By coupling these two isothermal techniques, this method effectively minimizes cross-reactivity among homologous miRNA targets and maximizes the sensitivity, achieving a LOD of 0.24 pM. As another case in point, utilization the high specificity of SDA, Gou et al. developed a novel linear displacement isothermal amplification (LDIA) method, which realize the specific detection of lethal infectious diseases (Figure 4h) (Gou et al., 2023). However, SDA’s dual-enzyme dependency elevates system complexity and introduces sensitivity to environmental pH fluctuations, representing ongoing challenges for its transition into fully autonomous POCT devices. Clinically, SDA is suited for precision diagnostics requiring rigorous specificity, such as discriminating homologous miRNA tumor markers or intricate pathogen typing (He et al., 2024). Conclusively, the dual-verification nature of the SDA process makes it a useful asset for mitigating cross-interference in complex biological matrices, prioritizing analytical accuracy over assay simplicity.

In conclusion, while each isothermal amplification technology possesses specific operational strengths and inherent technical limitations, their continued refinement and integration are expanding the capabilities of molecular POCT. Table 1 systematically compares the technical features, advantages, limitations, applicable scenarios, clinical translation progress, and additional key performance metrics of seven mainstream isothermal amplification methods.

**TABLE 1:** Isothermal amplification technology.

Technical name	Technical features	Advantages	Limitations	Turnaround time	Multiplexing capacity	Applicable scenarios	Clinical approval status
LAMP	4–6 specific primers, strand-displacing polymerase-mediated cyclic amplification, 60 °C–65 °C (De Felice et al., 2022)	High efficiency, rapid detection, no complex equipment, visualizable results (De Felice et al., 2022)	Complex primer design, prone to non-specific amplification, contamination risk (Liu et al., 2023)	15–30 min	Low–moderate (2–5 targets)	On-site pathogen detection, grassroots screening, food safety control (Liu et al., 2023)	Multiple diagnostic products cleared by FDA/NMPA/CE for influenza A virus, *Mycobacterium tuberculosis*, SARS-CoV-2, etc (De Felice et al., 2022)
RPA	Recombinase-primer recognition, SSB stabilization, 37 °C–42 °C isothermal amplification (Seder et al., 2023)	Mild temperature, rapid, anti-inhibition, minimal sample processing (Seder et al., 2023)	High reagent cost, contamination risk from lid removal, primer-dependent specificity (Hu et al., 2024)	15–25 min	Moderate (up to 30 with spatial coding	Emerging infectious disease testing, POCT, resource-constrained settings (Hu et al., 2024)	Multiple products granted Emergency Use Authorization (EUA) by CE/FDA for mpox virus, SARS-CoV-2, etc (Hu et al., 2024)
NASBA	RNA-specific, RNA→cDNA→RNA cycle, 41 °C, no thermal denaturation (Ju et al., 2021)	RNA-specific recognition, no denaturation, detectable RNA products (Kang et al., 2022)	Reverse transcriptase instability, complex system, inhibitor-sensitive (Ju et al., 2021)	15–60 min	Low	RNA virus detection, gene expression, miRNA analysis (Ju et al., 2021)	Commercialized products available for respiratory syncytial virus (RSV), HIV detection (Ju et al., 2021)
RCA	Circular probe template, DNA polymerase-mediated rolling replication, isothermal (Moon et al., 2022)	Ultra-strong signal amplification, high specificity, simple operation (Xu et al., 2023)	Circular probe dependency, secondary structures, long duration (Seder et al., 2023)	60–120 min	Low–moderate	Low-abundance target detection, SNP analysis, biomarker quantification (Moon et al., 2022)	Mainly in laboratory research stage, few products under development (Seder et al., 2023)
HDA	Helicase unwinding, SSB stabilization, 37 °C–42 °C, no denaturation (Qin et al., 2026)	No denaturation, simple operation, high primer-dependent specificity (Qin et al., 2026)	Helicase thermostability, high enzyme cost, sequence-dependent efficiency (Shen et al., 2023)	30–60 min	Low	Pathogen detection, clinical low-abundance screening, integrated POCT (Shen et al., 2023)	Few research-use only products, no large-scale clinical application (Qin et al., 2026)
CHA	Enzyme-free, hairpin probe cyclic assembly, 25 °C–37 °C isothermal (Zhao et al., 2023b)	Easy storage, mild conditions, high specificity, low background (Ma et al., 2026)	Precise probe design, target concentration dependency, needs combination for low abundance (Zhao et al., 2023b)	30–90 min	Moderate	Low-abundance signal amplification, infectious diseases, tumor markers (Ma et al., 2026)	In laboratory validation stage (Ma et al., 2026)
SDA	Restriction enzyme nicking, strand-displacing extension, 37 °C–41 °C cyclic (Liu et al., 2026)	High dual-recognition specificity, rapid, no thermal cycler (Mohammadniaei et al., 2021)	Dual enzyme dependency, pH-sensitive, minimal non-specific risk (Liu et al., 2026)	30–60 min	Low	miRNA testing, pathogen typing, clinical precision diagnosis	Exploratory applications in miRNA tumor marker detection (Mohammadniaei et al., 2021)

### 2.3 CRISPR/Cas systems: single-nucleotide resolution recognition

The CRISPR/Cas system has evolved from a prominent gene-editing tool into a highly versatile molecular diagnostic platform (Gootenberg et al., 2017; Chen et al., 2018). Its single-nucleotide target recognition, programmable crRNA design, and target-activated collateral cleavage activity provide robust specificity and potent signal amplification capabilities (Karlikow et al., 2023; Wachholz Junior and Kubota, 2024). Consequently, integrating the CRISPR/Cas system with upstream isothermal amplification has emerged as a leading strategy to overcome common analytical bottlenecks, particularly the insufficient sensitivity and non-specific cross-reactivity often associated with conventional isothermal methods (Siddiqui et al., 2025).

A microfluidic paper-based analytical device that couples RPA with CRISPR/Cas12a biosensing was developed for screening of cervical cancer (Figure 5a) (Liu S. et al., 2025). This approach leverages the stringent target recognition of Cas12a to verify specific amplicons, effectively mitigating false positives caused by non-specific pre-amplification. Additionally, Zhang et al. established a portable centrifugal microfluidic system that integrates nucleic acid extraction with multiplexed CRISPR/Cas13a detection (Figure 5b) (Zhang Y. et al., 2024). Requiring only a single sample loading step, this preloaded automated platform enables the simultaneous detection of up to 10 viruses within 45 min. Furthermore, A battery-powered M3-CRISPR platform was reported by Huang’s group, successfully combining RPA and CRISPR technology to enhance field applicability in resource-constrained settings (Figure 5c) (Zhou et al., 2023). Ultimately, the deep integration of CRISPR/Cas systems with isothermal pre-amplification substantially mitigates the long-standing trade-off between rapid, on-site operational convenience and rigorous diagnostic accuracy (Liu et al., 2025c).

**FIGURE 5:**
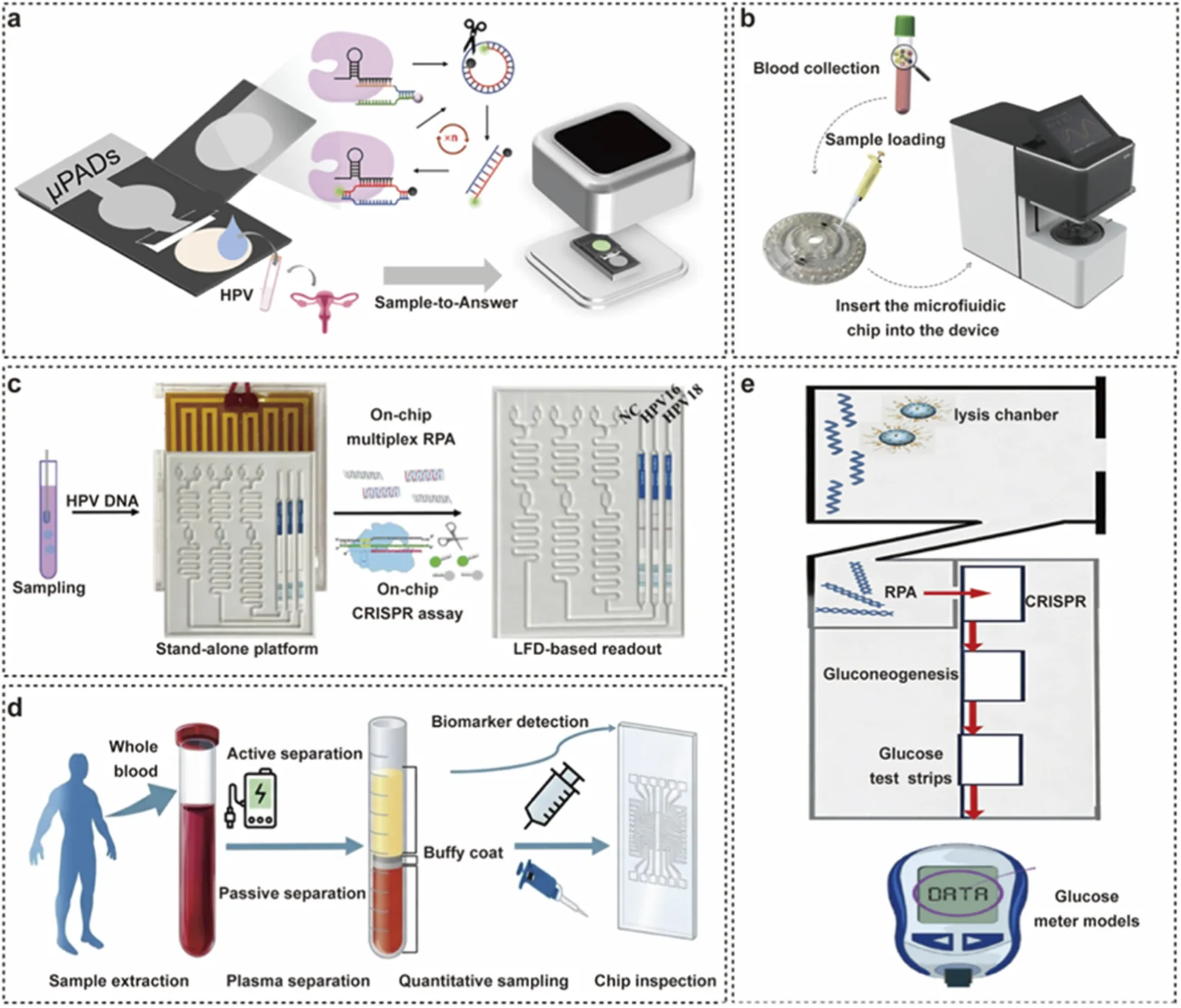
**(a)** The illustration of paper-based RPA-CRISPR/Cas12a biosensor for HPV detection (Liu S. et al., 2025). **(b)** Design and layout of the POCMT device and its corresponding microfluidic chip (Zhang Y. et al., 2024). **(c)** Schematic diagram of the M3-CRISPR detection platform (Zhou et al., 2023). **(d)** The illustration of the on-chip plasma separation module (Sun et al., 2025). **(e)** Structural configuration of a self-driven microfluidic sensor chip for Norovirus nucleic acid extraction and detection (Wang W. et al., 2026).

By introducing programmable crRNA recognition, CRISPR/Cas diagnostics address the limitation of non-specific cross-reactivity that frequently affects standalone isothermal methods. The primary advantage of integrating CRISPR with upstream pre-amplification is the attainment of single-nucleotide resolution, which enables PCR-level specificity without the need for thermal cycling instrumentation. Despite these capabilities, the transition of CRISPR diagnostics into widespread clinical use is hindered by unresolved challenges (Jung et al., 2024), such as the formulation complexity of its multi-component reagents (Cas proteins, crRNAs, and reporter probes) and the increased production costs. Additionally, improving the ambient stability of Cas enzymes and establishing standardized regulatory performance criteria remain necessary barriers to overcome. These platforms are well-suited for settings requiring the accurate discrimination of closely related pathogen strains or point mutations in clinical, particularly in decentralized environments. In summary, CRISPR/Cas integration resolves the traditional trade-off between operational simplicity and diagnostic accuracy, although reagent complexity and cost currently restrict most systems to the research prototype stage.

### 2.4 Sample preparation: the critical bottleneck for true sample-to-answer POCT

#### 2.4.1 Sample collection and storage

Commercial chemically-modified papers, such as Fast Technology Analysis (FTA) cards, facilitate the collection, transport, and ambient storage of blood, cells, and viruses. Beyond paper-based matrices, flocked nylon swabs enable efficient elution of pathogens from nasopharyngeal and oropharyngeal specimens, while capillary blood collection tubes and anticoagulant-coated microvials support standardized liquid sample acquisition with minimal training (Wilkinson et al., 2025). Magnetic bead-coated swabs further allow simultaneous collection and preliminary target capture, reducing downstream processing time. Self-sampling oral fluid collection devices and dried blood spot (DBS) microsampling cards extend accessibility to community-level and home-based testing. Vacuum-assisted saliva collectors and buccal swab kits preserve nucleic acid integrity through integrated lysis buffers, while microfluidic capillary chips with metering structures enable precise volume control directly at the point of collection. These diverse collection strategies collectively reduce reliance on specialized phlebotomy and cold-chain logistics, laying the foundation for decentralized molecular diagnostics.

#### 2.4.2 Sample separation

Raw clinical samples often require separation to isolate relevant components and prevent matrix interference with downstream detection signals. While paper-based membranes such as LF1 and Fusion 5 utilize capillary filtration to separate plasma from whole blood, centrifugal microfluidic discs exploit rotational forces for high-efficiency plasma separation with minimal sample volume. Acoustic standing-wave devices focus cells by density differences without physical filters, enabling gentle separation of fragile blood components. Dielectrophoretic separation leverages frequency-dependent dielectric properties to selectively trap or repel target cells, while immunomagnetic bead-based depletion uses antibody-functionalized particles to remove abundant interferents such as leukocytes or albumin. Cross-flow filtration membranes and inertial microfluidic channels achieve size-based particle sorting at high throughput, and hydrophoresis exploits hydrodynamic interactions in slanted ridge structures for label-free cell separation. These complementary approaches address the diverse separation demands of blood, saliva, urine, and environmental samples (Shi et al., 2026).

#### 2.4.3 Sample extraction

Isolating target nucleic acids or proteins typically involves complex multistep operations including lysis, binding, washing, and elution. Silica-coated magnetic beads and spin-column silica membranes remain benchmark methods for high-purity nucleic acid extraction, leveraging chaotropic salt-mediated binding and ethanol washing. Thermoplastic microfluidic chips fabricated by hot embossing or injection molding integrate all extraction steps within sealed channels, enabling closed-system processing that minimizes contamination risk. Glass fiber and polypropylene monolith columns provide alternative solid-phase supports with tunable pore sizes, while polymer-coated magnetic nanoparticles offer rapid magnetic separation without centrifugation (Fu et al., 2025). Paper microfluidic origami devices perform guanidinium thiocyanate-based DNA extraction through sequential folding, and surface acoustic wave (SAW) atomization has been combined with lateral-flow strips to extract proteins and cells using high-frequency mechanical vibration (Zhao et al., 2019). Solid-phase extraction cartridges with anion-exchange resins further enable selective capture of viral RNA from crude lysates, and automated liquid-handling workstations standardize extraction across large sample batches (Chen et al., 2026).

#### 2.4.4 Sample concentration

To detect analyte present at low concentrations, a concentration step is often required to improve assay sensitivity and reduce the limit of detection. Aqueous two-phase systems (ATPS) partition target biomarkers into specific liquid phases through differential solubility, enabling gentle concentration without membrane fouling. Ultrafiltration membranes and centrifugal concentration devices achieve volume reduction through size exclusion, while tangential-flow filtration supports continuous concentration of large sample volumes. Immunomagnetic separation selectively enriches target cells or nucleic acids using antibody- or aptamer-functionalized magnetic particles, offering high specificity even in complex matrices. On-chip isotachophoresis (ITP) focuses sample ions into contiguous zones between leading and trailing electrolytes, achieving up to thousand-fold concentration within minutes. Dielectrophoretic trapping and electrokinetic stacking further concentrate charged analytes at electrode interfaces, and centrifugal ultrafiltration in microfluidic discs enables rapid volume reduction for point-of-care applications. Chitosan-modified paper facilitates in-membrane sample purification through pH-dependent electrostatic interactions with nucleic acids (Sun et al., 2025).

## 3 Integrated technologies for POCT automation and accessibility

Molecular diagnostic technologies achieve their full potential when coupled with advanced integration platforms. Specifically, microfluidics and smartphone-based systems bridge the gap between complex biochemical reactions and user-friendly, decentralized deployment.

### 3.1 Microfluidics: high-throughput and miniaturized assays

POCT is frequently hindered by cumbersome manual operations, large volume requirements, and a heavy reliance on complex preprocessing equipment (Duan et al., 2024). To overcome these limitations, microfluidic technology manipulates fluids precisely through micron-scale channel networks (Whitesides, 2006; Haghayegh et al., 2024; Han et al., 2025). Offering remarkable miniaturization and high integration, this approach consolidates extraction, amplification, and detection into a single closed chip (Huang et al., 2024; Iyer et al., 2025; Jalali et al., 2026; Ma et al., 2018). As a prime example, Sun et al. developed an 8-channel, capillary-driven microfluidic chip for quantitative plasma separation (Figure 5d) (Sun et al., 2025). Capable of processing eight distinct 5 µL whole blood samples simultaneously, the device realizes a true “sample-in, answer-out” workflow. Taking a similar but modular approach, Wang et al. engineered a self-contained, compartmentalized device optimized for sample preprocessing and cascade reactions (Figure 5e) (Wang W. et al., 2026). Equipped with 12 parallel reaction units, the chip facilitates the simultaneous detection of norovirus across a dozen independent samples. Furthermore, being fabricated via cost-effective 3D printing, the system couples seamlessly with a commercial personal glucose meter for rapid signal readouts.

By integrating fluid manipulation into closed channels, microfluidics addresses the limitation of manual pipetting and reliance on bulky laboratory infrastructure, offering the advantage of automated, multi-step assay execution. However, a notable unresolved challenge is the manufacturing gap between academic prototyping (often utilizing PDMS or 3D printing) and commercial-scale injection molding, which frequently requires extensive redesign and optimization. Clinically, microfluidic integration is applicable for decentralized settings that require multiplexed, sample-to-answer processing without trained personnel. Thus, microfluidics bridges the gap between complex biochemical assays and portable deployment, while standardizing its manufacturing processes remains a necessary step for broader clinical translation.

### 3.2 Smartphones: intelligent analytical terminals

Despite rapid advancements, a significant number of POCT protocols remain tethered to specialized laboratory equipment and professionally trained personnel. Although integrating automated analysis modules can reduce human intervention, such additions inevitably inflate both the physical footprint and the structural complexity of these devices. Serving as an ideal countermeasure to these operational constraints, modern smartphones present a transformative solution. By natively incorporating high-resolution cameras, sophisticated sensors, wireless communication, and robust computational power, they effectively function as versatile, pocket-sized analytical hubs (Ozcan, 2014). Illustrating this potential, Ma et al. engineered a smartphone-controlled system paired with a passive, self-driven LAMP microfluidic chip for H1N1 influenza detection (Ma et al., 2019). In this specific configuration, a custom portable module captures real-time colorimetric signals, seamlessly transmitting them to the handset via Bluetooth. Taking an even more highly integrated approach, Zhao et al. constructed a portable smartphone platform featuring on-chip isothermal temperature control alongside droplet fluorescence imaging (Zhao et al., 2025). Coupled with a one-pot digital RPA/CRISPR assay, this system wholly circumvents the need for bulky commercial benchtop instruments. Consequently, it delivers a rapid, 50-min “sample-to-answer” quantitative readout while boasting an exceptional limit of detection of just 1 copy/µL. Further expanding on this mobile integration, Fozouni et al. developed an amplification-free CRISPR-Cas13a assay for the direct detection of SARS-CoV-2 RNA utilizing a mobile phone-based fluorescence microscope (Fozouni et al., 2021). By combining multiple crRNAs to maximize Cas13a target recognition and directly quantifying viral load through enzyme kinetics, this portable platform entirely eliminates the need for standard nucleic acid pre-amplification. Remarkably, the device achieves a sensitivity of approximately 100 copies/µL within 30 min of measurement and successfully identifies positive clinical nasal swab samples in under 5 min.

Leveraging consumer electronics, smartphone-based readout systems address the limitation of relying on specialized optical sensors, offering the advantage of decentralized data processing and connectivity. Nevertheless, unresolved challenges persist, including the variability in camera sensors across different smartphone models that necessitates device-specific calibration, as well as data privacy concerns for cloud-based processing. Clinically, smartphone integration is applicable for community-level screening and home-use diagnostics where accessibility is prioritized over laboratory-level throughput. In conclusion, smartphones function as versatile analytical hubs that lower the barrier to POCT, though standardizing readout consistency across different hardware platforms remains an ongoing technical requirement.

## 4 Diverse clinical applications

Driven by synergistic innovations like LAMP, RPA, CRISPR/Cas systems, and microfluidic technologies, molecular POCT has successfully overcome its reliance on specialized centralized laboratories. This technological framework now provides crucial technical support across diverse clinical scenarios.

### 4.1 Infectious disease containment via rapid pathogen identification

POCT has significant advantages in the rapid detection of infectious diseases. POCT can address these stringent requirements, particularly for highly mutable viruses such as HIV-1, which drive frequent treatment failures in resource-limited settings (Nicholas et al., 2019). A CRISPR-Cas12a-mediated surface-enhanced Raman scattering (SERS) lateral flow assay was developed for HIV detection (Figure 6a) (Pang et al., 2022). Leveraging the single-nucleotide specificity of Cas12a (detailed in Section 2.3), the assay accurately detects the HIV-1 M184V drug-resistant mutation at an allele frequency as low as 0.01%, achieving an ultrasensitive limit of detection of 0.3 fM without target pre-amplification. For the rapid diagnosis of respiratory infections with POCT (Kuitunen and Renko, 2023), a thermally activated one-pot RPA-CRISPR-Cas12b assay named TRACER was constructed by Feng et al. for *Mycoplasma* pneumoniae detection (Figure 6b) (Feng et al., 2025). Building on the isothermal kinetics of RPA and the collateral cleavage activity of Cas12b, the system enables streamlined one-tube testing with minimal instrumentation (K et al., 2022).

**FIGURE 6:**
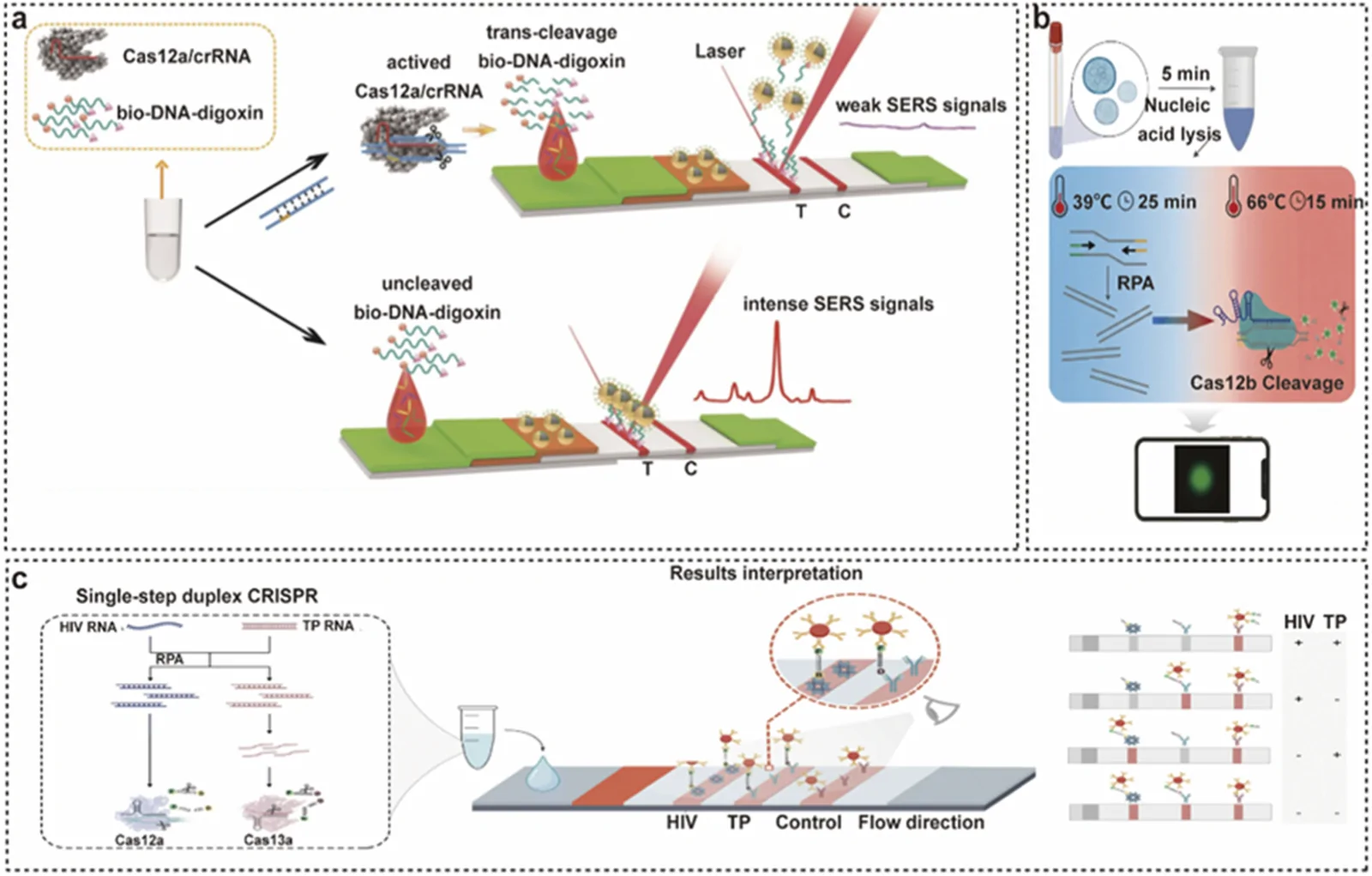
**(a)** Working principle of the Cas12a-SERS test strip for the detection of target dsDNA (Pang et al., 2022). **(b)** The TRACER platform for *M. pneumoniae* detection (Feng et al., 2025). **(c)** Schematic illustration of the DIAL assay for the simultaneous detection of HIV and *Treponema pallidum* (Xu et al., 2026).

To address the clinical need for simultaneous HIV and *Treponema pallidum* (TP) co-infection screening, Xu et al. engineered the DIAL platform (Figure 6c) (Xu et al., 2026). This system integrates reverse transcription, multiplex isothermal amplification, and dual Cas12a/Cas13a detection in a single one-pot reaction, paired with an engineered “line-elimination” lateral flow strip for intuitive, instrument-free visual readout within 45 min. Rapid POCT address the limitation of delayed centralized testing, providing the advantage of timely pathogen identification to facilitate clinical intervention (Diallo et al., 2021; Gaudenzi et al., 2020). An ongoing challenge in this field is the transition from single-pathogen detection to syndromic testing panels (Szlachta-McGinn et al., 2022) that can simultaneously differentiate multiple infections with overlapping symptoms (Lim et al., 2024) without compromising assay simplicity. Clinically, these platforms are applicable in emergency triage and remote clinics during infectious disease outbreaks (Cavuto et al., 2025; Fan et al., 2026). Overall, molecular POCT supports infectious disease containment by providing actionable results at the point of need (Bloemen et al., 2023), while expanding multiplexing capacity remains a primary developmental focus.

### 4.2 Early oncology screening via ultra-sensitive detection

To overcome these analytical barriers, Yang et al. developed a highly sensitive fluorescent biosensor on a paper-based microfluidic chip (see Section 3.1 for microfluidic design principles) for the precise detection of the EGFR exon 19 deletion mutation in ctDNA (Cai et al., 2021; Yang et al., 2024). Using MBene nanosheets for efficient DNA adsorption, the platform amplifies fluorescence signal output while enabling testing with minimally invasive peripheral blood samples.

For cervical cancer screening in low-resource settings, Gao et al. established the RotEx-LAMP-LFA system for the detection of HPV E6/E7 oncogenic transcripts (Gao et al., 2025). Built on the robust isothermal amplification performance of LAMP, the platform enables field-deployable molecular triage without thermal cycling equipment. Integration with RT-RAA pre-amplification, a CRISPR-Cas9-based lateral flow strip was developed by Liu’s group for dual-gene (PCA3/KLK3) detection of prostate cancer (Chen et al., 2023). The instrument-free platform achieves high analytical sensitivity, and the PCA3/KLK3 signal ratio reliably differentiates prostate cancer from benign prostatic hyperplasia in clinical specimens. As a single-cartridge diagnostic tool, the assay delivers high-sensitivity results within 45 min, supporting accessible community-based screening programs.

Decentralized oncology screening addresses the limitation of relying on centralized sequencing infrastructure, providing the advantage of localized, minimally invasive liquid biopsy analysis (Xu C. et al., 2022). However, a significant unresolved challenge is ensuring sufficient analytical sensitivity to detect low-abundance circulating tumor DNA (ctDNA) in early-stage cancers (Bartolomucci et al., 2025; Wang et al., 2024), alongside the need for clinical validation in large patient cohorts. Clinically, these assays are suitable for primary care and community health centers aiming to facilitate early cancer detection (Bhaskar et al., 2026). In summary, while molecular POCT enables broader access to oncology screening, enhancing sensitivity and securing regulatory validation remain necessary for routine clinical adoption.

### 4.3 Cardiovascular emergency triage via multi-parameter integration

Characterized by rapid progression and the necessity for “minute-level” clinical assessment to initiate life-saving interventions, cardiovascular conditions such as acute myocardial infarction (AMI) demand extreme diagnostic urgency (Park, 2020). Because traditional testing paradigms rely heavily on centralized laboratories, they suffer from notoriously time-consuming workflows that frequently miss optimal therapeutic windows. To bridge this critical temporal gap, Li et al. developed a capillary-based microfluidic chip explicitly engineered for rapid AMI detection (Figure 7a) (Li et al., 2023). Capable of simultaneously quantifying three core AMI biomarkers, this platform achieves an impressive limit of detection of 0.1 ng/mL for cardiac troponin I (cTnI), thus enabling the precise identification of early-stage myocardial injury. Following a similar trajectory to optimize POCT for multiplexed AMI biomarkers, Yuan et al. engineered an integrated, self-driven microfluidic immunofluorescence chip for the simultaneous quantification of myoglobin (Myo), creatine kinase-MB (CKMB), and cTnI (Figure 7b) (Yuan et al., 2024). By utilizing ultrasonic atomization and spray technology to apply a carboxyl-functionalized silane coating, they successfully modified the polymethyl methacrylate (PMMA) chip surface, which significantly enhanced both fluidic self-driving capabilities and robust antibody immobilization. This streamlined platform completes multi-target detection within 10–15 min and demonstrates exceptional analytical sensitivity, including a broad linear response for cTnI down to 0.05 ng/mL. Pushing the boundaries of point-of-care accessibility even further, Wang et al. reported a 3D affinity biosensor tailored for pre-hospital and home-based AMI screening (Figure 7c) (Wang Z. et al., 2022). By evaluating biomarkers directly within unprocessed whole blood in a mere 5 min, this system effectively circumvents the need for cumbersome sample pre-processing steps.

**FIGURE 7:**
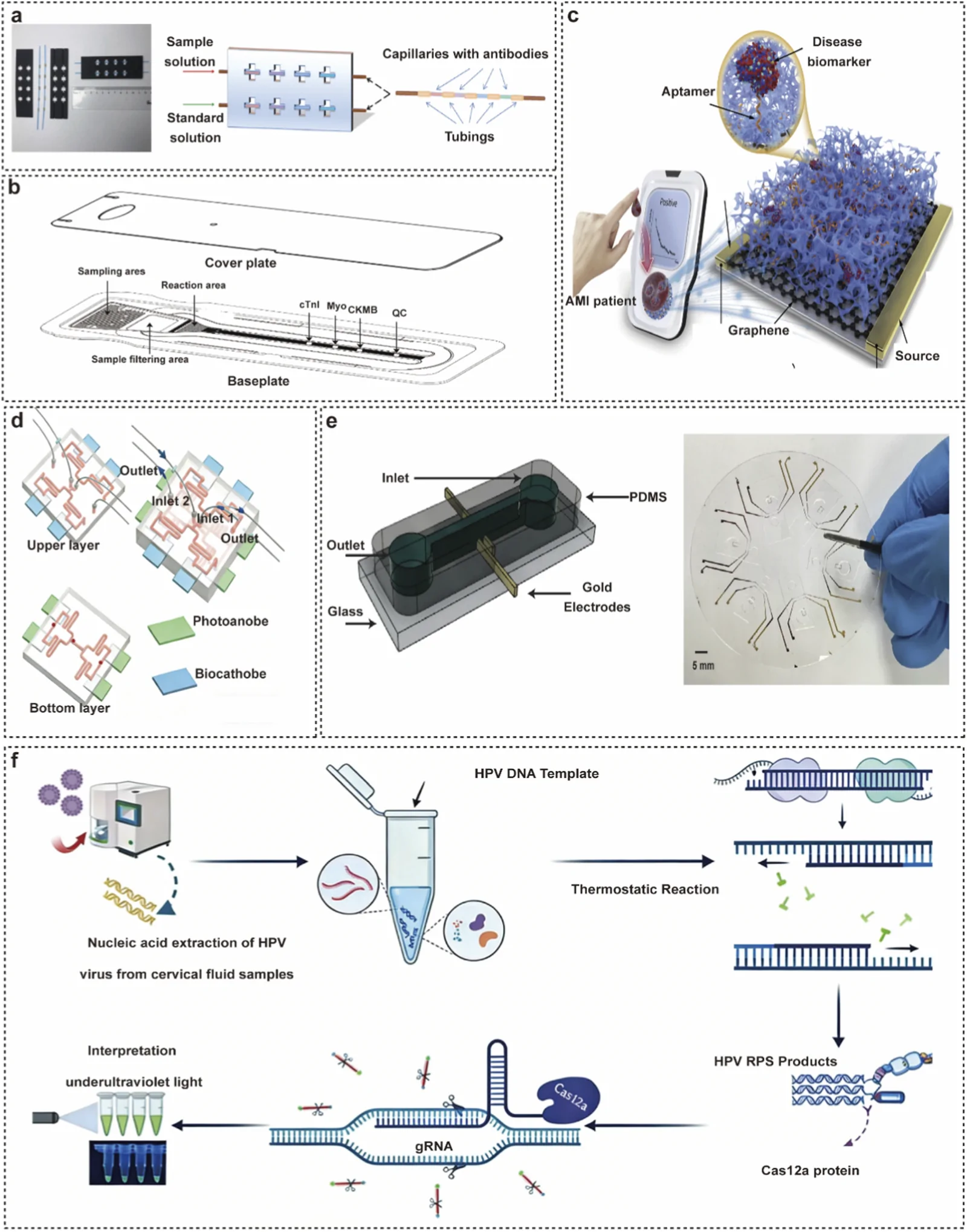
**(a)** Schematic design and operation of a capillary-based microfluidic chip for multiplex biomarker detection (Li et al., 2023). **(b)** Structural composition and layout of the microfluidic chip (Yuan et al., 2024). **(c)** The illustration of the 3DA-biosensor for AMI biomarker detection (Wang Z. et al., 2022). **(d)** Schematic representation of the fabricated microfluidic chip (Li J. et al., 2022). **(e)** The microfluidic chip for transthyretin amyloidosis genotyping via multifrequency impedance spectroscopy (Tayyab et al., 2025). **(f)** The schematic for the nucleic acid extraction process and the HPV-multiple RPA-CRISPR/Cas12a (H-MRC12a) typing assay (Liu et al., 2024).

By enabling rapid, on-site biomarker quantification, cardiovascular POCT addresses the limitation of prolonged laboratory turnaround times that often exceed the therapeutic window for acute events. A key advantage is the capacity to simultaneously assess multiplexed cardiac panels, facilitating swift emergency triage. Nonetheless, advancing these systems toward continuous, wearable monitoring before patients seek medical care presents unresolved challenges in sensor stability and calibration. Clinically, these tools are highly applicable in emergency departments and pre-hospital settings such as ambulances. Conclusively, multi-parameter POCT accelerates cardiovascular emergency care, with future developments directed toward continuous prognostic monitoring.

### 4.4 Metabolic disease management via continuous monitoring

For point-of-care genetic diagnostics, Tayyab et al. developed a label-free microfluidic impedance cytometry platform for allele-specific genotyping of transthyretin amyloidosis (Figure 7e) (Tayyab et al., 2025). By quantifying multifrequency impedance responses of solution-phase DNA, the device rapidly identifies pathogenic variants without the need for lengthy electrophoresis workflows.

Extending POCT applications beyond conventional metabolites to epigenetic biomarkers, Lu et al. combined CRISPR-Cas12a with RCA for the detection of miR-183, a key circulating marker of diabetic retinopathy (Lu et al., 2024). Building on the isothermal signal amplification properties of RCA (Section 2.2.4) and the target specificity of Cas12a, the assay achieves attomolar-level sensitivity without thermal cycling instrumentation. Decentralized metabolic testing addresses the limitation of infrequent, centralized laboratory check-ups, offering the advantage of routine, localized monitoring for chronic conditions (Li J. et al., 2022). The transition toward multi-omic monitoring, integrating genetic, epigenetic, and protein biomarkers (Song et al., 2023), poses unresolved technical challenges regarding device complexity and data interpretation. Clinically, these platforms are applicable for the management of chronic metabolic diseases in outpatient or home settings. To summarize, molecular POCT supports proactive metabolic disease management by enabling frequent biomarker evaluation, though simplifying multi-omic integration remains an ongoing objective.

### 4.5 Personalized health management via proactive surveillance

For example, the rapid quantification of 17β-estradiol (E2) is of high clinical value for monitoring endocrine function. To enable point-of-care E2 testing, Jia et al. developed an ultrasensitive biosensing platform that integrates Cas12a collateral cleavage with a dual-cycle amplification strategy (Jia et al., 2025). The assay converts molecular recognition events into visible readouts using commercially available pregnancy test strips, enabling fully instrument-free detection.

Turning to infectious disease screening, timely detection of high-risk human papillomavirus (HR-HPV), the primary cause of cervical cancer, requires robust, decentralized testing solutions. To address this need, Liu et al. developed the H-MRC12a POCT system for genotype-specific HPV screening outside centralized laboratories (Figure 7f) (Liu et al., 2024). The platform combines multiplex RPA with Cas12a-mediated signal transduction, enabling robust detection of six major high - risk HPV genotypes within 40 min and bypassing the logistical constraints of conventional PCR-based screening.

Personalized health management devices address the limitation of reactive medical models, providing the advantage of proactive, decentralized surveillance for reproductive health and specific chronic markers. Unresolved challenges primarily involve establishing appropriate regulatory frameworks for over-the-counter (OTC) molecular tests, ensuring user - friendly design, and maintaining quality control in non-professional environments. For the clinical application, these systems are targeted toward home-use and community pharmacies to support independent health monitoring. In conclusion, molecular POCT broadens the scope of personalized health management, but widespread adoption relies on overcoming usability and regulatory barriers.

## 5 Conclusion and outlook

Over decades of technological iteration, molecular POCT has evolved from simple rapid test strips into a highly complex, multidimensional technological ecosystem. The current mainstream paradigm is well-established. Isothermal amplification eliminates the reliance on bulky thermal cyclers. CRISPR/Cas systems elevate target recognition to single-nucleotide resolution. Concurrently, microfluidic technology orchestrates these biochemical reactions into fully automated, “sample-in, answer-out” platforms. This technological synergy has successfully transitioned complex molecular diagnostics from central laboratories to the patient’s bedside. Consequently, it has greatly advanced the accessibility of precision medicine.

However, achieving the widespread adoption and true decentralization of molecular POCT requires overcoming several critical challenges from the “bench” to the “bedside”. In the coming period of time, the field of molecular POCT is poised to undergo a fundamental paradigm shift, moving far beyond the current phase of proof-of-concept demonstration of novel biosensing mechanisms. Instead, future development trajectories will converge on building fully integrated, environmentally sustainable, and artificially intelligent diagnostic ecosystems that systematically address the long-standing bottlenecks hindering clinical translation and industrial scale-up.

Thus far, most research advances in this area have concentrated on the design of novel molecular-recognition and signal-amplification chemistries, which are typically validated under ideal buffer conditions without accounting for practical constraints inherent to routine clinical workflows. This emphasis on the performance of individual components sustains a pronounced translational gap. Specifically, numerous high-performance laboratory assays fail to maintain their diagnostic sensitivity and specificity when challenged with crude clinical specimens; alternatively, they rely on labor-intensive manual procedures and specialized instrumentation that preclude their deployment in POCT. In the near future, research priorities will shift from optimizing isolated reaction steps to co-designing complete end-to-end diagnostic systems that integrate sample lysis, nucleic acid purification, target amplification, signal detection, and result interpretation into a single closed workflow. This integrated design philosophy will minimize hands-on user steps, reduce operator-induced variability, and ensure consistent, reliable assay performance across diverse care settings, from primary care clinics and emergency departments to community screening sites and home-based self-testing, even when operated by personnel without formal laboratory training.

Alongside functional integration, environmental sustainability will transition from a peripheral consideration to a core design pillar of next-generation diagnostic ecosystems. Conventional POCT relies heavily on single-use petroleum-derived plastic consumables, cold-chain reagent distribution, and centralized manufacturing, all of which carry substantial environmental costs and limit accessibility in resource-limited regions. Future platforms will be engineered for full life-cycle environmental responsibility: they will adopt biodegradable paper-based substrates or recyclable modular hardware components, incorporate ambient-stable lyophilized reagent formulations to eliminate cold-chain dependencies, and be compatible with localized on-demand manufacturing models that reduce the carbon footprint of long-distance global supply chains. This approach will not only reduce the ecological footprint of diagnostic testing but also advance health equity by expanding access to regions lacking robust laboratory and logistics infrastructure.

Finally, deep integration of artificial intelligence will transform these systems from passive detection tools into active, context-aware clinical decision support platforms. Moving well beyond basic smartphone-based signal readout and data storage, AI algorithms will be embedded across the entire diagnostic workflow. Upstream, machine learning models will automate sample quality assessment and correct for background matrix interference in unprocessed specimens, reducing false positive and negative results caused by suboptimal sample input. Downstream, clinically trained AI models will synthesize multiplex biomarker profiles with patient electronic health records, demographic data, and longitudinal testing history to generate stratified risk assessments, differential diagnostic insights, and personalized intervention recommendations. When coupled with emerging wearable molecular sensing technologies, these intelligent ecosystems will drive a structural shift from episodic, single-point testing to continuous, real-time health monitoring, enabling pre-symptomatic disease detection and dynamic, data-driven therapeutic management.

This holistic, ecosystem-level approach will also directly tackle key industrialization barriers that have historically slowed mass market adoption. Standardized modular cartridge designs, validated for high-throughput industrial manufacturing, will bridge the long-standing yield and cost gaps between academic prototyping and commercial scale production. In parallel, evolving regulatory frameworks tailored to integrated diagnostic systems will streamline clinical validation and market clearance pathways, accelerating the translation of innovative technologies from the bench to routine patient care. In sum, the next decade of molecular POCT development will be defined not by the number of novel sensing mechanisms reported, but by the ability to deliver robust, affordable, and equitable diagnostic solutions that integrate seamlessly into global healthcare systems.

### 5.1 Enhancing quality control and overcoming matrix effects

Existing platforms often exhibit ultra-low limits of detection in ideal buffer systems. However, unprocessed clinical samples (e.g., whole blood, saliva, and feces) introduce severe matrix effects in real-world settings (Liu Q. et al., 2025; Shang et al., 2023). These effects frequently lead to enzyme inactivation or significant background interference. To mitigate this, future POCT systems must integrate robust internal controls (ICs) and automated calibration modules within their microfluidic or paper-based architectures. Establishing robust internal quality control (IQC) systems to minimize false positive and false negative results is essential to ensure diagnostic reliability. Only then can molecular POCT earn the trust of clinicians and meet the rigorous reliability standards of precision medicine.

### 5.2 Overcoming scale-up bottlenecks and regulatory barriers

The industrialization of POCT currently faces a critical translational gap between laboratory prototyping and commercial manufacturing (Rei Yan et al., 2020). Many laboratory-scale microfluidic prototypes heavily rely on soft lithography using PDMS or rapid 3D printing. However, when transitioning to industrial-grade injection molding for mass production, these devices often suffer from plummeting yields, altered fluidic behaviors, and escalating costs. To bridge this gap, future developments must draw insights from successful industry paradigms. Commercial milestones, such as the Abbott ID NOW (isothermal amplification) and Cepheid GeneXpert (automated PCR) platforms, have successfully circumvented these physical bottlenecks by adopting highly standardized, modular cartridge designs. Furthermore, crossing manufacturing barriers requires engineering core enzymes (such as Cas effectors and helicases) for extreme ambient stability using synthetic biology and protein engineering. Industry leaders have widely adopted advanced lyophilization (freeze-drying) techniques to preserve enzyme integrity outside strict cold chains, a strategy that must be integrated earlier in academic prototype designs.

Beyond manufacturing, the commercialization of these technologies is severely hindered by a lack of extensive clinical validation data, compounded by the absence of a harmonized regulatory framework tailored specifically for decentralized molecular testing. This is particularly challenging for over-the-counter (OTC) home-use tests. Nevertheless, the regulatory adaptability witnessed during the COVID-19 pandemic “fool-proof” usability and quality control standards mandated by regulatory agencies. Ultimately, deep collaboration among bioengineers, regulatory bodies, and industrial manufacturers is indispensable to translate laboratory proof-of-concept into globally accessible healthcare products.

### 5.3 Transitioning to “deep intelligence” and continuous monitoring

Integrating molecular POCT technology into a broader digital health ecosystem will become an important direction for the development of POCT in the next decade (Wang X. et al., 2022; Zhang Q. et al., 2026). Currently, most smartphone-coupled POCT platforms serve primarily as portable readout terminals, limited to result visualization and basic data storage rather than active clinical decision support. Next-generation diagnostic frameworks will embed artificial intelligence (AI) across the entire testing workflow (Gao and Li, 2024): upstream, machine learning algorithms will automate sample quality assessment and correct for matrix interference and background noise in unprocessed specimens, reducing false readouts caused by suboptimal sample input; downstream, clinically trained AI models will integrate multiplex biomarker profiles with patient electronic health records, historical test trends, and demographic data to deliver stratified risk assessments, differential diagnostic suggestions, and personalized intervention recommendations (Topol, 2019).

Multiplex panels, particularly for chronic cardiovascular and metabolic diseases, will be tightly coupled with cloud-based computing architectures. Rather than processing individual test results in isolation, these systems will synthesize longitudinal diagnostic data over time to identify subtle, pre-symptomatic biomarker trajectories that would be undetectable in single-point measurements. For example, in diabetes management, AI-integrated POCT will enable dynamic adjustment of insulin dosing based on real-time glucose trends combined with circulating genetic and metabolic markers, moving far beyond static reference range-based interpretation.

Concurrently, the disease management paradigm is expected to shift fundamentally from episodic, single-point testing toward continuous, unobtrusive health monitoring. This transition will be enabled by advances in wearable molecular sensing technologies, including flexible epidermal biosensors, minimally invasive microneedle arrays, and interstitial fluid sampling platforms. Unlike conventional single-use POCT devices that require manual sample collection, these systems will enable autonomous, real-time tracking of circulating nucleic acids, proteins, and small-molecule biomarkers in sweat, interstitial fluid, or capillary blood, eliminating the diagnostic gaps between discrete testing events.

This transition toward continuous, AI-augmented molecular monitoring carries profound clinical implications: it can facilitate pre-symptomatic identification of acute events including myocardial infarction and early-stage infection, enable real-time therapeutic drug monitoring for precision oncology and chronic disease management, and underpin closed-loop drug-delivery systems capable of autonomously tuning dosages according to real-time biomarker readouts. At a population level, aggregated, anonymized POCT data streams will also strengthen real-time public health surveillance, enabling faster detection of pathogen outbreaks and more targeted deployment of public health resources. Realizing this full potential will, however, require addressing outstanding challenges including algorithmic generalizability across diverse patient populations, regulatory pathways for AI-augmented diagnostic devices, and standardized frameworks for secure, privacy-preserving health data sharing.

### 5.4 Moving toward sustainable “green POCT”

As molecular POCT continues to scale globally, particularly across low- and middle-income countries (LMICs) where centralized laboratory infrastructure remains scarce, the cumulative environmental footprint of disposable diagnostic consumables has emerged as an unavoidable, system-level challenge. Conventional POCT devices rely heavily on petroleum-based plastics for microfluidic chips, single-use cartridges, and packaging, alongside embedded electronic components that contribute to medical e-waste streams; the global cold chain required for reagent storage and distribution further adds substantial associated carbon emissions. Looking ahead to the next decade, the development of next-generation sustainable POCT will require a holistic, life-cycle-oriented approach to environmental impact, rather than incremental adjustments to individual components.

First, next-generation diagnostic carriers will transition from fossil-fuel-derived polymers to renewable, biodegradable substrates engineered to preserve analytical performance. Advanced functional paper-based materials, for instance, will not only serve as reaction supports but also integrate built-in sample filtration, plasma separation, and reagent storage capabilities, eliminating the need for additional plastic processing components. Beyond paper, bioplastics derived from renewable biomass (such as polylactic acid and alginate-based hydrogels) and natural fiber matrices will be optimized for microfluidic channel fabrication, balancing precise fluidic control, biocompatibility, and controlled biodegradability. This shift will reduce persistent plastic waste accumulation in clinical and community settings, particularly in regions without formal medical waste disposal systems.

Second, modular and circular design principles will redefine POCT product architectures to minimize total material waste. Instead of fully disposable all-in-one devices, future platforms will separate single-use, sample-contacting reaction modules from reusable electronic readout and control units. The reaction modules, fabricated from biodegradable materials, will be safely discarded after use, while the core detection hardware can be reused across thousands of tests. Refillable reagent reservoirs and replaceable assay cartridges will further extend device lifespans, substantially reducing the material footprint per individual test.

Third, sustainable manufacturing and distributed production models will reduce the carbon footprint associated with global supply chains. Traditional centralized manufacturing and long-distance refrigerated shipping account for a substantial share of POCT’s total life-cycle environmental impact. Moving forward, localized production via low-cost 3D printing and roll-to-roll paper processing will enable on-demand manufacturing of diagnostic substrates near points of care. This not only cuts transportation emissions and packaging waste but also improves supply chain resilience in LMICs, aligning environmental sustainability goals with improved global health accessibility.

Fourth, energy-free and passive detection systems will eliminate operational power demands and reduce reliance on cold chains. Instrument-free assays driven entirely by capillary action, spontaneous exothermic reactions, or passive wicking will enable testing without external power sources, making deployments compatible with off-grid settings while removing energy-related carbon costs. Coupled with advanced lyophilized reagent formulations that maintain stability at ambient temperatures for months, these systems will eliminate the need for continuous cold storage and transport, one of the largest energy burdens in current diagnostic supply chains.

Importantly, the Green POCT framework is designed to advance, rather than compromise, global health equity. Sustainable design choices must not come at the cost of diagnostic performance or affordability; instead, they should simultaneously reduce environmental harm and expand access by lowering production and distribution costs. In this sense, green innovation is not merely an environmental priority but a core enabler of equitable, resilient point-of-care diagnostics worldwide (Wang et al., 2025).

The ultimate vision of molecular POCT extends far beyond the creation of portable detection instruments. It aims to build a globally accessible diagnostic ecosystem that is faster, more precise, cost-effective, and intelligently interconnected - one that moves beyond reactive disease detection to enable proactive, personalized health management. Through sustained cross-sector collaboration between academia, industry, regulatory bodies, and global health organizations, continued advances in biomaterial engineering, scalable manufacturing, and adaptive regulatory frameworks will position molecular POCT as a foundational pillar of modern healthcare. Beyond individual patient care, this ecosystem will strengthen real-time global public health surveillance networks, enable early outbreak response, reduce health disparities across resource settings, and advance universal health equity for populations worldwide.

### 5.5 Wearable molecular sensing

As a foundational technology enabling the shift from episodic point-of-care testing to continuous health monitoring, wearable molecular sensing is redefining the boundaries of molecular POCT by delivering real-time, minimally invasive or non-invasive tracking of circulating biomarkers. Unlike conventional single-use POCT devices that generate discrete, single-time data points, wearable biosensors integrate molecular recognition components with flexible, skin-conforming substrates to enable sustained sampling and detection of target analytes from accessible biological matrices, including sweat, interstitial fluid (ISF), and tear fluid (Kim et al., 2019). This paradigm extends the clinical utility of molecular diagnostics beyond clinic-based testing to ambulatory, home, and field settings, aligning with the broader vision of proactive, personalized health management.

Technologically, wearable molecular sensing platforms fall into three primary categories based on sampling strategy. First, non-invasive epidermal biosensors leverage naturally secreted biofluids, most commonly sweat, to detect small-molecule metabolites, proteins, and even nucleic acid targets. Engineered flexible substrates functionalized with aptamers, antibodies, or CRISPR-based recognition elements enable continuous measurement of analytes such as glucose, lactate, inflammatory cytokines, and viral antigens directly on the skin surface. Second, minimally invasive microneedle arrays and reverse iontophoresis platforms access interstitial fluid just beneath the epidermis, providing access to circulating biomarkers that correlate more closely with blood concentrations, including circulating tumor DNA (ctDNA), microRNAs, and cardiac troponins. These systems can capture high-molecular-weight targets that are not readily present in sweat, closing the gap between non-invasive wearables and gold-standard blood testing. Third, textile-integrated and epidermal patch sensors embed microfluidic channels and reagent storage directly into flexible materials, enabling automated sample collection, on-board amplification, and signal readout without external user intervention.

The clinical applications of wearable molecular sensing span multiple disease domains. In chronic metabolic disease management, next-generation wearables will move beyond single-analyte continuous glucose monitoring to simultaneous tracking of multiple metabolic and genetic biomarkers, enabling AI-driven dynamic adjustment of insulin and other pharmacotherapies as described in Section 5.3. In cardiovascular care, continuous wearable monitoring of cardiac troponin I and B-type natriuretic peptide will enable pre-symptomatic detection of acute myocardial infarction and heart failure decompensation, addressing the critical time gaps inherent in episodic emergency department testing. In infectious disease control, wearable pathogen sensors will support real-time community-level surveillance during outbreaks, enabling rapid identification of infected individuals without centralized testing infrastructure. Additionally, wearable therapeutic drug monitoring will enable real-time dosing optimization for narrow-therapeutic-window medications such as chemotherapeutics and immunosuppressants, advancing precision medicine delivery.

Despite this promise, several technical barriers remain before wearable molecular sensing can achieve widespread clinical adoption. Long-term sensor stability remains a core challenge: biofouling from skin proteins and continuous exposure to biological matrices can degrade recognition elements over time, leading to signal drift and reduced accuracy. Non-invasive sampling strategies also face fundamental challenges in correlating biomarker concentrations in sweat or ISF to systemic blood levels, requiring robust calibration algorithms and population-specific validation. Additionally, integrating sensitive molecular amplification chemistries such as isothermal amplification into flexible, low-power wearable form factors remains technically demanding, particularly for low-abundance nucleic acid targets.

Looking forward, advances in antifouling biomaterials, flexible microfluidics, and low-power signal transduction will drive the next-generation of wearable molecular POCT devices. When integrated with cloud-based AI analytics and closed-loop drug delivery systems, these platforms will transform molecular diagnostics from a reactive, clinic-based service into a continuous, proactive component of everyday health management.

### 5.6 Decentralized manufacturing

The global deployment of molecular POCT has long been constrained by a centralized manufacturing model that relies on long-distance cold chain logistics, complex global supply chains, and bulk production cycles. This structure creates systemic vulnerabilities, including supply chain disruptions during public health emergencies, high transportation-related carbon footprints, and prohibitive costs for low- and middle-income countries (LMICs). Decentralized manufacturing, defined as localized, on-demand production of diagnostic devices and reagents close to points of care, emerges as a transformative solution that aligns with both the sustainability goals of Green POCT and the global health equity agenda.

Three core technological pillars are enabling decentralized manufacturing of molecular POCT platforms. First, low-cost desktop 3D printing technologies allow for on-site fabrication of microfluidic chips, assay cartridges, and device housings directly in clinical settings, field hospitals, or local production hubs. Unlike traditional injection molding, which requires high upfront tooling costs and large production runs, additive manufacturing enables small-batch, customized production with minimal lead times, making it ideal for outbreak response and resource-limited settings. Second, roll-to-roll processing of paper-based diagnostic substrates can be deployed at regional scale using simplified printing and coating equipment. Paper-based LAMP, lateral flow assay, and CRISPR detection platforms are particularly well suited to localized manufacturing, as their core materials are widely available and their production does not require cleanroom-level infrastructure. Third, *in-situ* reagent formulation and lyophilization capabilities enable local preparation and stabilization of assay master mixes, reducing reliance on long-distance cold chain shipping of temperature-sensitive enzymes and nucleic acid probes.

Decentralized manufacturing delivers multiple interconnected benefits across environmental, clinical, and health equity dimensions. Environmentally, it eliminates the carbon emissions associated with long-haul refrigerated transport and excess packaging, directly advancing the Green POCT framework. Clinically, it enables rapid, customized response to local disease burdens, and regional production hubs can rapidly develop and deploy diagnostic panels tailored to locally prevalent pathogens or disease phenotypes, rather than relying on one-size-fits-all products designed for global markets. From a health systems perspective, decentralized manufacturing strengthens supply chain resilience, reducing vulnerability to global disruptions such as pandemics, trade restrictions, and shipping delays. For LMICs in particular, local production reduces import costs and currency dependencies, making molecular POCT more affordable and accessible.

However, scaling decentralized manufacturing faces significant regulatory and quality control challenges. Ensuring consistent, reproducible assay performance across distributed production sites requires standardized quality control protocols, validated production workflows, and regular proficiency testing, all of which can be difficult to implement in low-resource settings. Regulatory frameworks also lag behind the technology: most medical device regulations are designed for centralized, large-scale manufacturing, and there is a lack of clear pathways for locally produced diagnostic tests to meet clinical safety and efficacy requirements. Additionally, core raw materials such as high-purity polymerases, Cas proteins, and modified oligonucleotides still rely on global supply chains, limiting the full independence of local production.

As regulatory frameworks evolve and production technologies become more accessible, decentralized manufacturing will become an increasingly integral component of the global molecular POCT ecosystem. By combining local production capacity with centralized quality oversight and standardized design templates, the field can achieve both environmental sustainability and equitable global access, ensuring that advanced molecular diagnostics are available whenever and wherever they are needed.
